# Cold-stored platelets: A systematic review of recovery in healthy adults and chest drain output in cardiothoracic surgery patients

**DOI:** 10.1016/j.htct.2025.106226

**Published:** 2025-12-12

**Authors:** Caleb Keane, Heba Sharif, Denise Jackson

**Affiliations:** Thrombosis and Vascular Diseases Laboratory, School of Health and Biomedical Sciences, RMIT University, Bundoora, Victoria, Australia

**Keywords:** Blood platelets, Cold-stored platelets, Platelet transfusion, Room-temperature platelets, Cardiothoracic surgery, Chest drain output, In vivo recovery, Activated platelets, Meta-analysis

## Abstract

Cold-stored platelets were abandoned in the 1960s after demonstration of an increased clearance in vivo due to an irreversible activated phenotype. Difficulties in storage, logistics, and the increased requirement of therapeutic platelet transfusions for haemostasis have sparked renewed interest in cold-stored platelets. This systematic review compared two primary outcomes: in vivo recovery for autologous cold-stored platelets *versus* room-temperature platelets in healthy volunteers, and chest drain output at 24 h for allogeneic cold-stored platelets *versus* room-temperature platelets after complex cardiothoracic surgery. A total of 4215 articles were found in the ProQuest, PubMed, Scopus, Embase, and Cochrane electronic databases. Seven eligible papers were included in this meta-analysis. Cold-stored platelets showed a decreased in vivo recovery two hours after retransfusion following storage for two to seven days compared to a room-temperature platelet control group (mean difference: −25.85 %; 95 % confidence interval: −41.98 to −9.71 %; p-value = 0.002). Further, cold-stored platelets showed a decreased chest cavity output when transfused within 24 h after complex cardiothoracic surgery (mean difference: 249.68 mL; 95 % confidence interval: 85.68 to 413.67 mL; p-value = 0.003). While cold-stored platelets are not a substitute for room-temperature platelets in a prophylactic scenario, their ability to significantly reduce chest cavity output suggests they may be optimal for the management of bleeding in surgical patients, especially in the context of logistical difficulties.

## Introduction

### A brief history of the platelet

Platelet concentrates serve an important function in transfusion medicine today, but as late as 1910, the role of platelets in haemorrhages was only just being described [1]. Duke observed platelets modulating haemorrhage in transfusions, and went on to suggest some haemorrhagic diseases were likely caused by extremely low platelet counts. However, it was not until the 1950s that the transfusion of platelets was systemically approached [2]. Like other therapeutic products, platelets were stored cold, and they were often used for haematological disorders, so they required a long lifespan in vivo [3]. Transfusion of these platelets demonstrated a nearly 50 % decrease in haemorrhage-related leukaemia fatalities, which sparked much research interest [4]. During the 1950s, research began to demonstrate that overall survival of cold-stored platelets (CSPs) was poorer than room-temperature platelets (RTPs), and the switch to plastic containers in the 1960s allowed better storage conditions for studies to demonstrate this phenomenon [2,5,6]. Given the desired outcome for most platelet transfusions was a sustained increment of patient platelet count, in the 1960s it was strongly suggested that the switch from CSPs to RPTs be made, and the entire industry quickly followed this recommendation [6].

### Platelet function and the platelet storage lesion

Platelets are small cytoplasmic fragments from bone marrow megakaryocytes that play several roles in haemostasis, thrombosis, and immune modulation [7]. In circulation, they are in a quiescent, thin disc shape. As thermosensors, they are primed at peripheral sites with lower temperatures, a process that induces the release of intracellular granules and biochemicals for subsequent activation [5,7, 8, 9]. In particular, α-granules containing P-selectin (CD62P), Platelet Factor 4 (PF4), von Willebrand factor (vWF), and CD63 are released and either bind to the surface of platelets to modulate their function or signal the immune system [10, 11, 12, 13]. They also undergo conformational changes in surface receptors, such as the fibrinogen receptor GPIIb/IIIa involved in aggregation, and the vWF receptor GPIb/IX/V involved in adhesion [14,15]. Activated platelets will remodel their actin filaments to form filopodia, which increase the surface area and allow better aggregation, in conjunction with degranulation [16, 17, 18].

The platelet storage lesion (PSL) is defined as any deterioration in platelet quality or viability that occurs during preparation and *ex vivo* storage [19]. PSLs manifest distinct effects that are dependent on the stage of component preparation. These effects are triggered by a range of factors including temperature fluctuations (*e.g.*, during whole blood transport), artificial surface contact, pathogen reduction technology, centrifugation forces, the storage media composition, and agitation (rocking) [19, 20, 21, 22]. PSLs include cellular activation, which leads to degranulation and biochemical release; cellular fragmentation, which results in decreased platelet count and reduced in vivo survival; and loss of functional receptors, which diminishes overall in vivo function [19, 20,23]. An additional problem of RTPs is bacterial contamination, which necessitates shorter storage times [20,21].

### Cold-Stored platelet troubles

Evidence in the 1960s led scientists to recommend room-temperature storage over cold-storage, due to the unique characteristics of platelets when exposed to colder temperatures. Platelets are thermosensors, and thus undergo various changes in metabolism, structure, and expression when they reach particular temperature thresholds [8,9]. Below 20 °C, platelets activate, which involves degranulation, increased surface expression of GPIIb/IIIa and P-selectin, and serotonin release [10,24,25]. Exposure to increased P-selectin levels, along with its associated receptor GPIb/IX/V, is a major component of the activated phenotype seen in CSPs [12]. Actin rearrangement leads to a spherical shape with pseudopods forming to increase surface area for aggregation [5,26,27]. The vWF receptors, GPIb, IX and GPV, are irreversibly clustered, with this condition being recognised by hepatic macrophage complement type 3 (CR3) receptors, leading to sequestration and phagocytosis in vivo [8,28,29]. Further, β-GlcNAc moieties on the cell surface are exposed through desialylation, leading to recognition and phagocytosis in the liver [30,31]. It has also been demonstrated that increased Ca^2+^ in storage, which may be an element of the storage medium, is correlated with increased aggregation in vivo [32]. These factors play a role in the observed increase in clearance and reduced survival of CSPs in vivo; studies which aim to demonstrate efficient function need to address these in vivo survival times and recovery levels to demonstrate efficacy of the product.

### Resurgence of cold storage interest

Despite many challenges, there has been an increase in both research and clinical interest in CSPs within the past two decades, due to both the changes in prophylactic platelet indications and clinical trial data. CSPs have several advantages compared to RTPs, and these may outweigh the drawbacks in conditions such as active bleeding, remote laboratories, or war zones [33,34]. CSPs demonstrate superior haemostatic function in vitro and in vivo, and refrigeration can reduce vasoactive substance release, leading to decreased febrile non-haemolytic transfusion reactions, and increased clearance, which can lower the risk of thrombosis [35, 36, 37]. Studies have demonstrated that CSPs maintain viability for up to 14 days in storage, which could markedly reduce wastage [38,39]. There is decreased metabolism and mitochondrial dysfunction, with less reactive oxygen species (ROSs) causing cellular damage [40, 41, 42]. CSPs have even been shown to better reverse anti-platelet drug-related bleeding [43]. One of the biggest advantages is the markedly decreased risk of bacterial sepsis, with refrigeration halting much bacterial overgrowth [44, 45, 46].

Use of CSPs would mitigate logistical challenges associated with transport of platelets, especially to remote areas, and in war zones [33]. Use of a dual inventory would allow platelets to be prescribed based on clinical appropriateness, and despite this being recommended in the 1970s when RTPs were universally instated, modern practices with prophylactic RTPs better support the use of a dual inventory [39,47]. A dual inventory study demonstrated the effectiveness of this design during the COVID-19 pandemic [39].

### Research aims

With the resurgence of interest in CSPs, there is limited evidence on the in vivo recovery of CSPs in the context of modern platelet preparation and in actively bleeding patients. Cardiothoracic surgery is common in the Western world, and platelet products better suited to these patients could lighten the burden on transfusion services and post-surgical interventions [38]. This study aims to compare current data using the PICO framework [48] by addressing the question: does transfusion of CSPs (intervention) in healthy volunteers (population) show variation in vivo recovery (outcome) when compared with RTPs (comparison)? A secondary question aims to address: does transfusion of CSPs (intervention) in cardiothoracic surgery patients (population) show a variation in chest cavity output (outcome) when compared with transfusion of RTPs (comparison)?

## Methods

### Search strategy

This systematic review and meta-analysis employed the Preferred Reporting Items for Systematic reviews and Meta-Analysis (PRISMA) protocol for study screening and eligibility assessment [49]. In addition, the Strengthening the Reporting of Observational Studies in Epidemiology (STROBE) checklist was applied to all full papers and conference abstracts to assess the quality of included studies [50]. To identify appropriate studies, searches were made of the PubMed, Cochrane, Scopus, Embase, and ProQuest electronic databases from inception date until July 2024. Searches used a combination of the following keywords: “cold-storage platelet”, “cold-stored platelet”, “thrombocytopenia”, “chilled platelet”, “room-temperature platelet”, “cardiac”, “malignancy”, “trauma”, and “cancer”. The ProQuest search employed additional restrictions: filtered by peer-reviewed, full-text only, and terms in abstract. Manually searching scientific databases did not turn up additional studies.

### Eligibility criteria

All articles were saved in Endnote automatically removing duplicates. The title and abstract of papers were screened and assessed for eligibility based on the research aim. The following criteria were assessed:

#### Types of studies

Observational studies, including both prospective and retrospective studies, were eligible for inclusion. Papers published in any time frame were eligible, and all journal articles were fully accessible and published in English. Papers must have had a minimum of two participants per study arm (*i.e.* CSP in vivo recovery, RTP in vivo recovery, CSP chest drain output, RTP chest drain output), and they must have measured both CSPs and RTPs for each cohort. Review articles and individual case studies were not included, but conference abstracts were acceptable.

#### Types of participants

For the primary research aim, studies analysing healthy individuals were considered eligible. Subjects must have been over 18 years old, not on any anti-platelet drugs, and have a normal platelet count. They must have consented to autologous collection of apheresis platelets stored in plasma, subsequent product treatment, and then their return for in vivo testing.

For the secondary research aim, studies analysing patients undergoing semi-urgent complex cardiothoracic surgery who had no history of congenital coagulopathies or haemostatic disorders, and who had not taken anti-platelet drugs within 48 h of the surgery, were considered. The patients must have received at least one unit of apheresis platelets stored in platelet additive solution (PAS) or PAS-C within 24 h after surgery without requiring reoperation. All patients must have consented during admission screening.

#### Types of outcomes measured

For the primary research aim, the papers must have included in vivo recovery for both CSPs and RTPs, with recovery presented as percentage of subject’s fresh autologous platelets two hours after reinfusion. Data must be presented as mean ± standard deviation (SD), or with standard error of mean (SEM) or 95 % confidence intervals (95 % CIs), from which SD was calculated. For the secondary research aim, the papers must have included the type of surgery, the chest drain output in mL 24 h post-surgery, and the corresponding data for both RTPs and CSPs. Studies were excluded if they did not measure both RTPs and CSPs.

### Data extraction

The following relevant data were extracted from eligible studies: primary author, year of publication, study design, study period (if indicated), country of origin, number of participants, participant population, cold-storage time, platelet type, percent in vivo recovery of RTPs, percent in vivo recovery of CSPs, and chest drain output 24 h post-surgery in mL for patients that received RTPs and those that received CSPs.

### Statistical analysis

Cochrane RevMan software was used to perform the meta-analyses [51]. A two-arm study of in vivo recovery of CSPs and RTPs was performed, as well as a two-arm study of chest drain output at 24 h after CSP or RTP treatment in cardiothoracic surgery patients. Each analysis used the inverse variance method measuring mean difference and employed the continuous effects analysis model presented as Forest plots. The software calculated statistical significance as p-value and 95 % CIs, with p-value <0.05 indicating statistical significance; and heterogeneity of studies as I^2^ with a corresponding p-value. The software was also used to calculate SDs for any studies that provided 95 % CIs or SEMs instead of SD. Risk of bias for included papers was assessed using Funnel plots generated by the software.

## Results

### Study selection

**Figure. 1:**
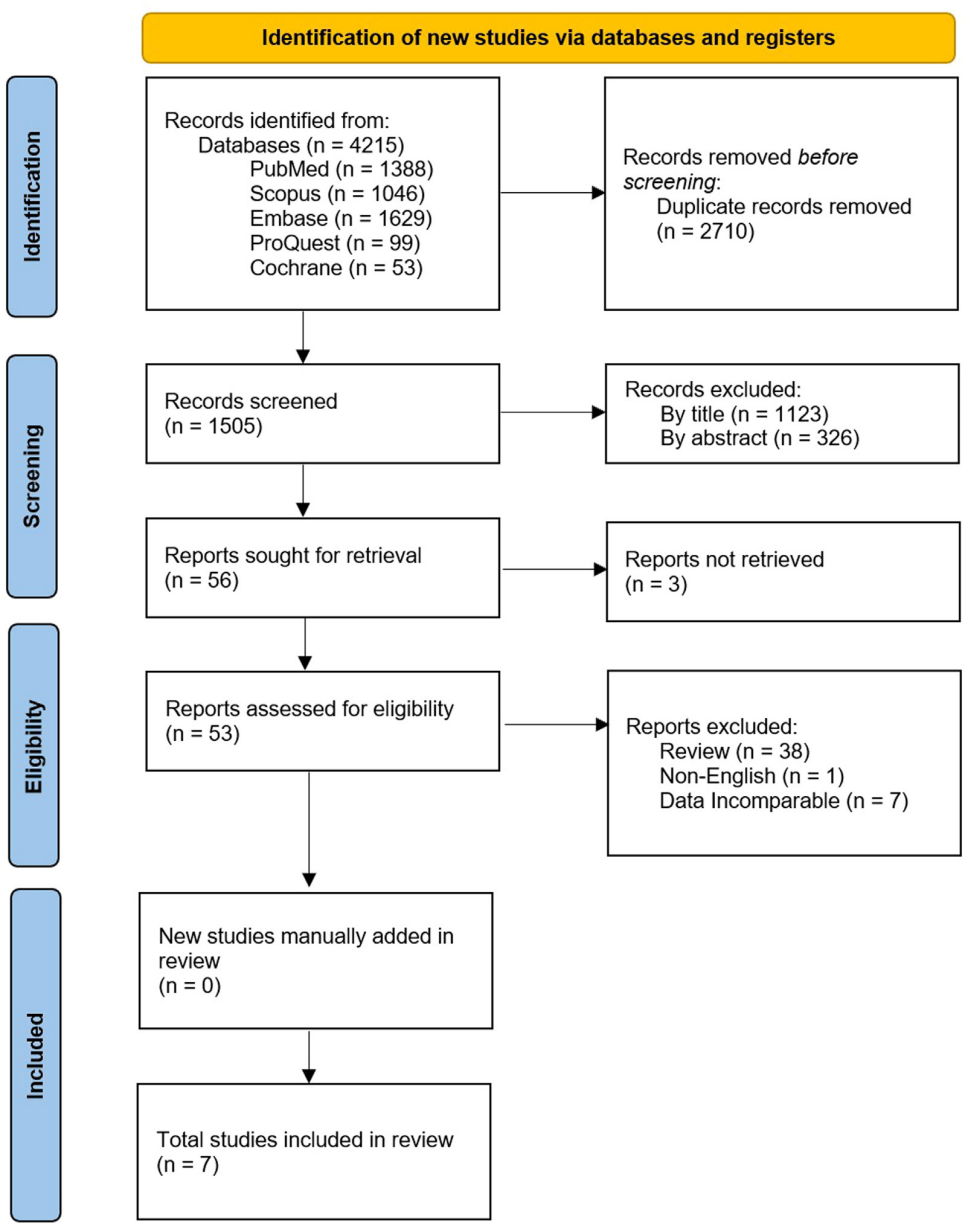
PRISMA flowchart for the identification and inclusion of relevant studies for the meta-analysis of in vivo recovery of transfused autologous platelets stored either at room temperature or refrigerated for two to seven days, and chest drain output 24 h after complex cardiothoracic surgery with transfusion of allogenic platelets stored either at room temperature or refrigerated [49].

The search strategy found 4215 articles published in electronic databases (PubMed, Scopus, Embase ProQuest, and Cochrane). EndNote was employed to remove 2710 duplicates before screening. Articles were screened first by title, removing a further 1123, as they were not relevant to the topic. Next, abstracts were screened based on eligibility and 326 were excluded after failing to meet the study criteria. Fifty-six full-length articles or conference abstracts were thoroughly assessed for eligibility. Three were removed after not being retrievable, 38 as they were review articles that did not present data, one for not being published in English, and then seven for having data that did not meet all the criteria for this meta-analysis. Therefore, seven articles were identified as eligible. A breakdown of the articles excluded in each stage of screening can be found in Figure. 1. The reference lists of the selected papers, as well as excluded review papers, were examined but no additional articles were identified. All seven identified papers were included in this meta-analysis.

### Study characteristics

The seven studies included in this review and meta-analysis measured the in vivo recovery of transfused autologous CSPs or RTPs two hours post-transfusion [31,53,54,56], or the chest drain output 24 h post-surgery for complex cardiothoracic surgery patients who received CSPs or RTPs [38,52,55]. All seven studies were prospective and conducted in the USA or Norway (Table 1). Four of the studies comprised healthy volunteers [31,53,54,56]; the other three consisted of cardiothoracic surgery patients [38,52,55].

**Table 1:** Characteristics of eligible studies investigating the in vivo recovery of transfused autologous platelets.

Study	Study Design	Study Period	Country	Number of Patients	Patient Population	Parameter Measured	Cold-Storage Time	Platelet Type
Apelseth et al. 2017 [52]	Prospective	Finished 2017	USA	35	Complex cardiothoracic surgery patients	RTP and CSP post-op chest drain	<7 days	Apheresis in PAS
Bailey et al. 2022 [53]	Prospective	Not indicated (but funding published in 2021)	USA	6	Healthy volunteers	RTP and CSP platelet in vivo recovery	5 days	Apheresis in plasma, apheresis in PAS-C
Stolla et al. 2020 [54]	Prospective	2016 – 2018	USA	5	Healthy volunteers	RTP and CSP platelet in vivo recovery	5 days	Apheresis in plasma
Strandenes et al. 2016 [55]	Prospective	Finished 2016	USA	26	Major cardiothoracic surgery patients	RTP and CSP post-op chest drain	<7 days	Apheresis in PAS
Strandeneset al. 2020 [38]	Prospective	2015 – 2018	Norway	50	Elective and semi-urgent cardiac surgery patients	RTP and CSP post-op chest drain	<7 days	Apheresis in PAS-C
Vostal et al. 2018 [56]	Prospective	Not indicated	USA	16	Healthy volunteers	RTP and CSP platelet in vivo recovery	7 days	Apheresis in plasma
Wandall et al. 2008 [31]	Prospective	Not indicated	USA	4	Healthy volunteers	RTP and CSP platelet in vivo recovery	2 days	Apheresis

RTP: Room-temperature platelets; CSP: Cold-stored platelets; PAS-C: platelet additive solution C (Intersol).

Platelets were stored either at room temperature or refrigerated for two to seven days and chest drain output was assessed 24 h after complex cardiothoracic surgery with transfusion of allogenic platelets stored either at room temperature or refrigerated.

The length of time platelets were stored cold before transfusion varied between the studies: two studies transfused after five days [53,54], one study transfused after two days [31], one study transfused after seven days [56], and all three chest drain output studies transfused within seven days but without stating a specific time [38,52,55]. All seven studies used platelets collected by apheresis, with in vivo recovery studies storing platelets in plasma [31,53,54,56] and chest drain output studies storing platelets in PAS or PAS-C [38,52,55].

All four in vivo recovery studies gave recovery as a mean percentage of the study arm ± SD (Table 2). For the three chest drain output studies, one reported mean output ± SD [55], one reported mean output ± SEM [52], and the other reported mean output with 95 % CI [38]. The SDs for papers that did not include them were calculated by RevMan software using the mean, SEM or 95 % CI, and population size. For Apelseth et al. the SD for CSPs was calculated to be 252 mL and for RTPs it was 511 mL [52]. For Strandenes et al. the SD for CSPs was calculated to be 327 mL and for RTPs it was 546 mL [38].

**Table 2:** Results of extracted data from eligible studies included in the meta-analyses.

Study	Patients Receiving RTP	Patients Receiving CSP	RTP in vivo Recovery%	CSP in vivo Recovery%	RTP Drain	CSP Drain
Apelseth et al. 2017 [52]	22	17	-	-	820 mL (109)⁎	546 mL (61)⁎
Bailey et al. 2022 [53]	21	5	92 ± 12	46 ± 7	-	-
Stolla et al. 2020 [54]	5	5	70 ± 7	46 ± 3	-	-
Strandenes et al. 2016 [55]	12	14	-	-	1055 mL ± 677 mL	775 mL ± 534 mL
Strandenes et al. 2020 [38]	25	25	-	-	865 mL (640–1091 mL)†	649 mL (514–784 mL)†
Vostal et al. 2018 [56]	12	4	55.7 ± 13.9	23.1 ± 8.8	-	-
Wandall et al. 2008 [31]	2	2	47 ± 13	53 ± 5	-	-

RTP: Room-temperature platelets; CSP: Cold-stored platelets; SEM: standard error of the mean; 95 % CI: 95 % confidence interval.

Results are given as mean ± SD unless otherwise indicated. The SEM and 95 % CI results are represented as they were found in the studies; however, calculated SDs for these papers are presented in the results section and in the meta-analyses.

⁎ Mean (SEM).

† Mean (95 % CI).

### Quality assessment of included studies

The quality, assessed for the seven studies using the STROBE checklist, is shown in Tables 3A & 3B for full-length articles and conference abstracts, respectively. All criteria except two were met by every study. In full-length articles, only one paper clearly stated the eligibility criteria of the included patients, setting of the study, and collection methods [38]. Also, only two full-length articles described the participant characteristics and possible confounders in the results section [31,38]. Both conference abstracts met all quality criteria and were considered high quality. The paper by Bailey et al. was a letter to the editor providing further data on a previous study and had no subsections, but the title was clear and the study design was indicated so it was considered eligible for the first criterion [53]. Stolla et al. reported potential biases in their results and not methods, so it was not considered ineligible for reporting of bias [54]. The paper by Wandall et al. did not have sample sizes large enough for statistical analysis (two participants per study arm), so it did not report statistical methods [31]. However, the study met the inclusion criterion of a minimum of two participants per study arm and thus it was included.

**Table 3A:** Evaluation of the methodology of included full-length studies.

	Title and Abstract	Introduction	Methods				Results	Discussion
	Clear title and abstract with study design indicated	Explain the scientific background and rationale	Study methods presented clearly	Eligibility criteria, setting, dates, and data collection described	Statistical methods described	Describes and addresses potential bias	Describes characteristics of study participants and potential confounders	Summarise key results and discusses limitations
Bailey et al. 2022 [53]	Y^a^	Y	Y	N	Y	Y	N	Y
Stolla et al. 2020 [54]	Y	Y	Y	N	Y	Y^b^	N	Y
Strandenes et al. 2020 [38]	Y	Y	Y	Y	Y	Y	Y	Y
Vostal et al. 2018 [56]	Y	Y	Y	N	Y	N	N	Y
Wandall et al. 2008 [31]	Y	Y	Y	N	N^c^	Y	Y	Y

Y: Criteria fulfilled; N: Criteria not fulfilled.

a Small study in letter to the editor format, no subsections like abstract.

b Addressed, but in the results section, not in the methods.

c Sample size sufficient for proof of hypothesis but not large enough for statistical analysis.

**Table 3B:** Evaluation of the methodology of included conference abstracts.

	Clear title with study design indicated	Study methods presented clearly	Eligibility criteria and setting briefly mentioned	Primary outcome of report clearly defined	Statistical methods described	Number of participants in study reported	Measures of variability or uncertainty reported	General interpretation of study given
Apelseth et al. 2017 [52]	Y	Y	Y	Y	Y	Y	Y	Y
Strandenes et al. 2016 [55]	Y	Y	Y	Y	Y	Y	Y	Y

Y: Criteria fulfilled; N: Criteria not fulfilled.

According to the Strengthening the Reporting of Observational Studies in Epidemiology (STROBE) checklist for full-length articles [50].

### In vivo recovery meta-analysis

**Figure. 2:**
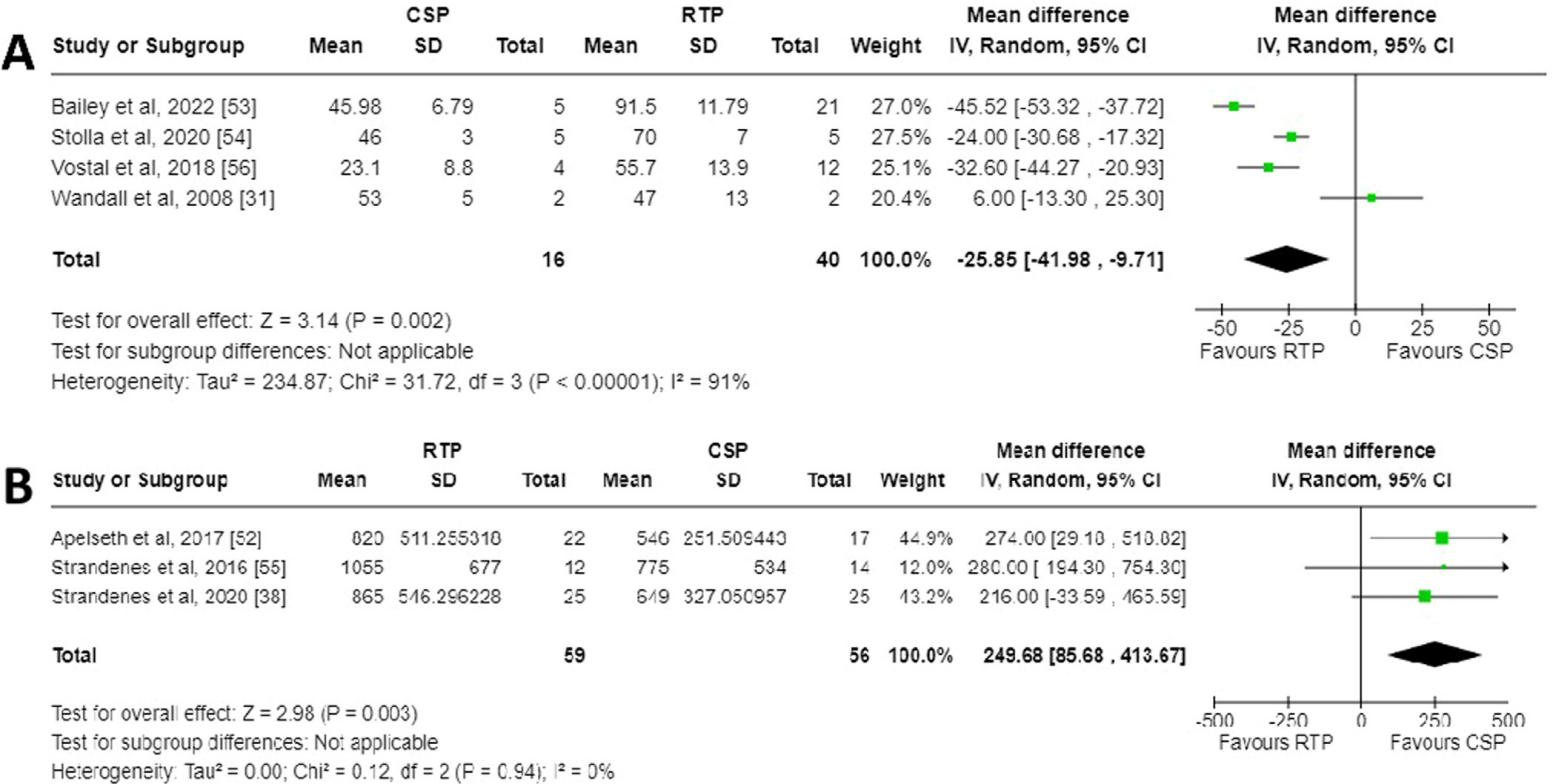
Forest plots for meta-analyses generated using RevMan [51]. **A)** Forest plot for in vivo recovery of transfused autologous platelets stored either at room temperature or refrigerated for two to seven days. **B)** Forest plot for chest drain output 24 h post complex cardiothoracic surgery with transfusion of allogenic platelets stored either at room temperature or refrigerated.

**Figure. 3:**
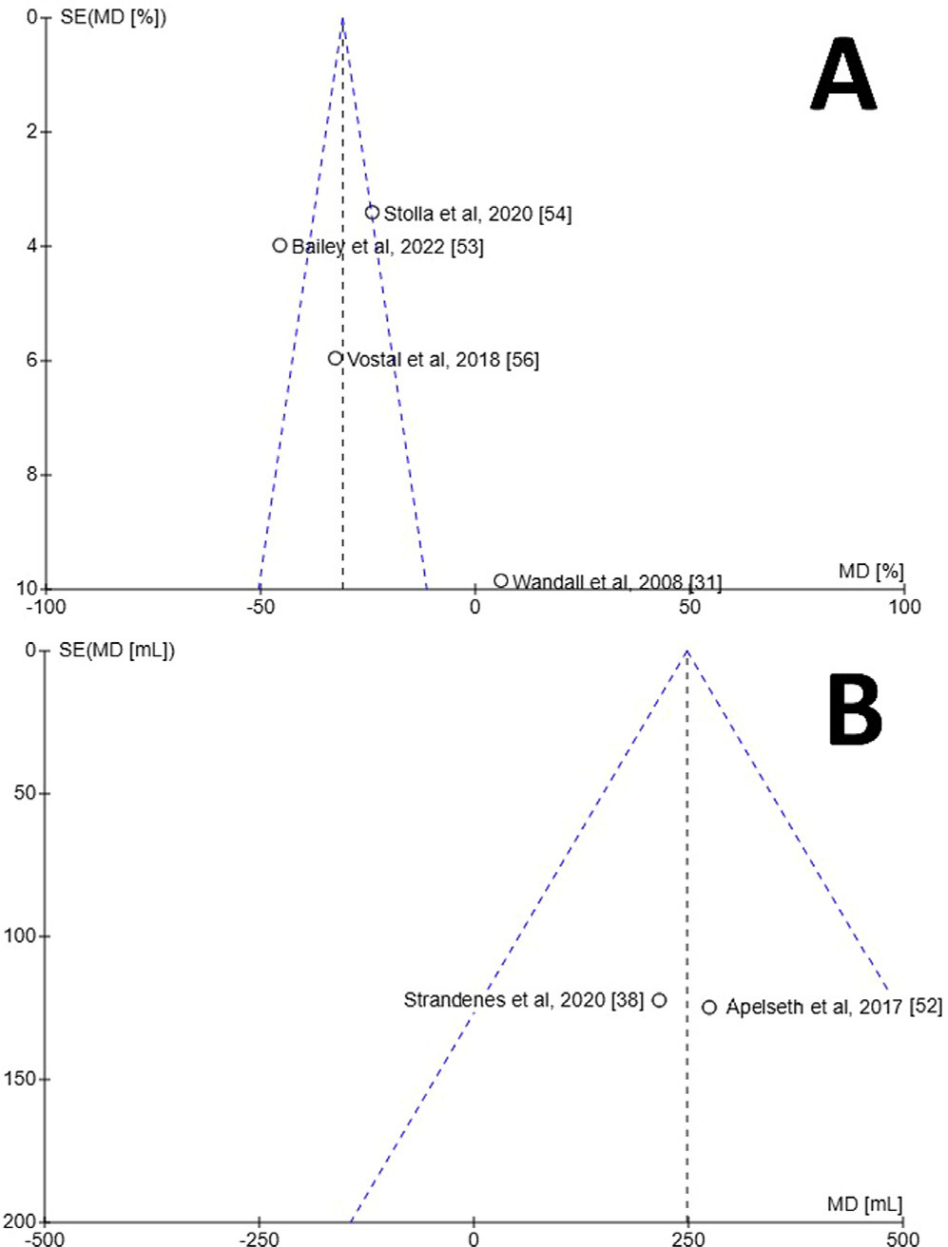
Funnel plots for estimation of bias generated using RevMan [51]. Programme did not generate 95 % confidence interval lines, likely due to small number of samples. **A)** Funnel plot for in vivo characteristics of transfused autologous platelets stored either at room temperature or refrigerated for two to seven days. **B)** Funnel plot for chest drain output 24 h after complex cardiothoracic surgery with transfusion of allogenic platelets stored either at room temperature or refrigerated. Programme did not display paper by Strandenes et al. [55].

A meta-analysis was performed and a Forest plot generated for the in vivo recovery of autologous CSPs and RTPs in healthy volunteers two hours after retransfusion (Figure. 2A). Across the four studies which reported in vivo recovery, three favoured RTPs for increased in vivo recovery whereas one favoured CSPs [31]. The mean difference between CSPs as a study group and RTPs as a control group was −25.85 % (95 % CI: −41.98 to −9.71 %), showing overall favour for RTPs. This finding was deemed statistically significant with a p-value of 0.002. The data between the studies demonstrated high heterogeneity (I^2^ = 91 %; p-value <0.00001). Risk of bias was assessed using a Funnel plot (Figure. 3A). Only four studies were included, so estimation of intervention effect is hard to determine from plot symmetry. The paper by Wandall et al. demonstrated the highest SEM and the greatest variation in mean difference between studies [31].

### Chest drain output meta-analysis

A meta-analysis was performed and a Forest plot generated for the chest drain output 24 h post-surgery after treatment with CSPs or RTPs (Figure. 2B). Across the three studies which reported chest drain output, all favoured CSPs for decreased output. The mean difference between CSPs as a study group and RTPs as a control group was 249.68 mL (95 % CI: 85.68 to 413.67 mL), overall, in favour of CSPs. This finding was deemed statistically significant with a p-value of 0.003. The data between studies demonstrated no heterogeneity (I^2^ = 0 %; p-value = 0.94), which likely indicates the studies are homogenous, and any differences in values are probably due to random sampling errors. Risk of bias was assessed using a Funnel plot (Figure. 3B). Only three studies were included, so estimation of intervention effect is hard to determine from plot symmetry. The paper by Strandenes et al. cannot be seen on the graph as the software was unable to place it in any viewable area, a fact that could not be mitigated with RevMan software [55].

## Discussion

### Reduced recovery of cold-stored platelet transfusion in vivo

This systematic review with meta-analysis shows that current studies are still in agreement with the long-understood decrease in vivo recovery of autologous platelets when stored cold before retransfusion. The overall recovery for CSPs was statistically significantly lower than RTPs, with a mean difference of −25.85 % (95 % CI: −41.98 to −9.71 %; p-value = 0.002). There was high heterogeneity of the data (I^2^ = 91 %; p-value <0.00001), which indicates that 91 % of the variation in results cannot be attributed to chance alone but is the result of other factors such as bias [57]. This heterogeneity may be high due to the small number of included studies, the small number of participants in each study arm, and reported patient-to-patient variations in many of the included studies. Since 2003, the Food and Drug Administration of the United States (FDA) have required platelet in vivo recovery to be ≥66 % [58], and CSPs did not meet this criterion in any study (53 ± 5 % [31], 46 ± 7 % [53], 46 ± 3 % [54], and 23 ± 9 % [56]).

Though beyond the scope of this review, all included papers also tested in vitro parameters to measure the activated phenotype observed in cold storage. This consists of assessing surface receptors such as CD62P (P-selectin) and phospholipid phosphatidylserine (PS), which are stored intracellularly and released during activation or in response to temperature drops; along with metabolic markers such as glucose and lactate, which are consumed to supply the energy needed for shape changes but are often considerably decreased at room temperature [11,12,31,38,40,53,54]. While there are increased activation marker levels in CSPs, including P-selectin and annexin V binding (an indirect test of PS expression), metabolic markers and pH are decreased, which may allow for better storage times and product quality.

One of the included studies showed slightly increased recovery for CSPs, likely due to the very short cold storage time (48 h) compared to the five- or seven-days others were stored [31]. While only demonstrated in one study, a shorter cold storage time displaying greater in vivo recovery may be a further avenue of research. Irreversible cold storage lesions usually occur after 18 h of storage at cold temperatures and so they can be ameliorated by warming within this period [6,59]. Combining cold storage with room temperature storage in a temperature cycling pattern therefore may yield better recovery and metabolic results. Vostal et al. studied the effects of temperature cycling, but only the data on CSPs was used for this meta-analysis [56]. They also reported that the study arms with CSPs and RTPs were not performed on the same participants, were performed at different times due to funding issues, used two different manufacturers for the collection of apheresis platelets, and thus demonstrated up to 10 % variation in results between study arms, which undoubtedly contributed to the heterogeneity seen in this meta-analysis.

Two of the studies found unexpected results when adapting their models from previous animal studies, which showed better outcomes than were reported in humans [31,56]. Differences in both metabolic markers and in vivo parameters demonstrated that structures and functions exist in human platelets which contributed to clearance of platelets; they were not seen in animal studies. This suggests that animal platelets are not a good substitute for human platelet testing, a major reason animal studies were excluded from the scope of this analysis.

### Reduced chest cavity output in cold-stored platelet transfusion

This systematic review with meta-analysis shows that transfusion of CSPs results in a lower chest cavity output within 24 h after complex cardiothoracic surgery compared with RTPs. The chest drain output in CSP transfusion was statistically significantly lower, with an overall mean difference of 249.68 mL (95 % CI: 85.68 to 413.67 mL; p-value = 0.003). There was no heterogeneity in the data (I^2^ = 0 %; p-value = 0.94), indicating that all the variation in the results is due to chance and not bias [57]. Despite overall findings showing significance when transfusing CSPs, each individual study did not report significance between participant groups. Both individual studies reporting no significant difference between groups and this meta-analysis reporting significant decrease in chest cavity output for CSPs indicate that CSPs are a suitable substitute for RTPs in the setting of acute bleeding in complex cardiothoracic surgeries. This is the logical conclusion for the transfusion of a haemostatically superior product in the context of haemostasis.

All platelet products employed PAS or PAS-C as the storage media, which has a reported lower incidence of transfusion reactions, and is routinely used for RTP storage. PAS-C has been demonstrated to show better in vivo recovery and survival, potentially influencing the decreased output compared to platelets in plasma used for the in vivo recovery meta-analysis [60]. All three included studies were conducted by mostly the same research team, which may have been part of the reason that no heterogeneity was seen in the data. There are ongoing trials in Australia and the USA using CSPs in surgical and bleeding patients, but data had not been published at the time of this study. The data from these clinical trials will be very useful in further demonstrating the effects of CSPs in halting bleeding.

A major problem with the design of these studies is the non-specific nature of chest drain output as a marker of haemostasis in surgery patients. In 2020, Strandenes et al. reported five different types of cardiac surgery with approximately the same number in each study arm [38]. Factors such as history of sternotomies, length of surgical procedure, patient age, logistic EuroSCORE, and patient blood volume all impact the required number of platelets required for restoration of haemostasis, as well as expected blood loss. These factors are very difficult to control for, and chest cavity output may not represent effectivity of platelets. Additionally, platelets were rarely given in isolation, but along with other transfusion products, and these products undoubtedly impacted blood loss. While this review points to a significant lowering of chest drain output, the most important finding is that CSPs currently demonstrate acceptable testing parameters for use during complex cardiothoracic surgery. Given the logistical difficulties of RTP storage and transport, they may be a viable option for some hospitals and centres.

### Limitations of review

The major limitation of this review was the small number of papers included, and the small number in each study arm in the papers. Only 56 healthy patients were tested across four studies for in vivo recovery, split between CSPs (*n* = 16) and RTPs (*n* = 40), and only 115 cardiothoracic surgery patients were tested across three studies for chest cavity output, split between CSPs (*n* = 56) and RTPs (*n* = 59). High heterogeneity for in vivo recovery was likely due to the size of these studies. Failure to test for both CSPs and RTPs, or differences in testing parameters, accounted for the exclusion of otherwise acceptable studies. Another important marker of in vivo platelet function is survival, which could not be tested as insufficient papers reported comparable results, with some reporting survival in days and others as a percentage of fresh count. Three of the in vivo recovery papers reported CSPs as a control for modified platelets stored cold, and therefore did not address problems with cold storage but with the modification of the platelets [31,53,56].

### Further research

CSPs in plasma have shown in vivo recovery levels lower than FDA requirements, but this meta-analysis did not report on the addition of other storage media or modifications to platelets, which may increase circulation time [58]. Research has demonstrated the various mechanisms by which CSPs are cleared rapidly from circulation, and continued research into stopping irreversible cold storage changes, such as galactosylation of clustered GPIb/IX receptors, is one avenue just starting to be reported in human trials; further research comparing these methods to a control group may lead to better preparation methods to promote cold storage [31]. Modification of platelets will be dependent on patient treatment. Increased recovery and survival are a requirement of prophylactic platelet treatment, but may also increase the effects of therapeutic platelets by virtue of increased time in circulation to participate in haemostasis. However, therapeutic platelets benefit from priming as they are effective immediately upon transfusion.

Research into storage conditions, such as temperature cycling (which may mitigate irreversible changes to platelet structure), is required to better understand the effects on storage time, in vivo recovery and survival, and product viability. Results from this research could elucidate new ways of storing platelets, allowing dual inventories or switching to an entirely new storage method. Two clinical trials are looking at how extended storage of CSPs for cardiac surgery may impact bleeding [61,62]. Purpose-built refrigerators can automate the storage process, reducing waste and increasing product effectiveness. Another clinical trial is determining efficacy of autologous CSPs that are reinfused after platelet count is normalised, in a similar fashion to acute normovolaemic haemodilution methods [63]. CSPs are only just being reintroduced for the treatment of bleeding, but their potential is still being understood even today, and future research needs to address the different ways platelets can be stored and modified to allow longer shelf life, better therapeutic function and life, and reduced risk to the patient.

## Conclusion

This systematic review and meta-analyses demonstrates that CSPs will have a statistically significantly reduced in vivo recovery when stored cold for two to seven days and tested two hours after retransfusion. This is due to an activated phenotype which occurs when they are stored below 20 °C for over 18 h, and recognised by hepatic macrophages which quickly clear platelets from circulation. Therefore, CSPs are not an effective prophylactic replacement for RTPs. Because of this activated phenotype, CSPs demonstrated a statistically significantly lower chest drain output when transfused within 24 h after complex cardiothoracic surgery. Ongoing clinical trials will hopefully provide further data to demonstrate increased haemostatic effectiveness of CSPs for therapeutic transfusion.

## Funding

This research did not receive any specific grant from funding agencies in the public, commercial, or not-for-profit sectors

## Data availability statement

The data used to support this study's findings are included within the article.

## Conflicts of interest

Authors declare no conflict of interest.
